# Supplementary figures and images for: In Vivo Analysis of Trypanosoma cruzi Persistence Foci at Single-Cell Resolution
Source: mBio. 2020 Aug 4;11(4):e01242-20. doi: 10.1128/mBio.01242-20 (PMC7407085; doi:10.1128/mBio.01242-20)

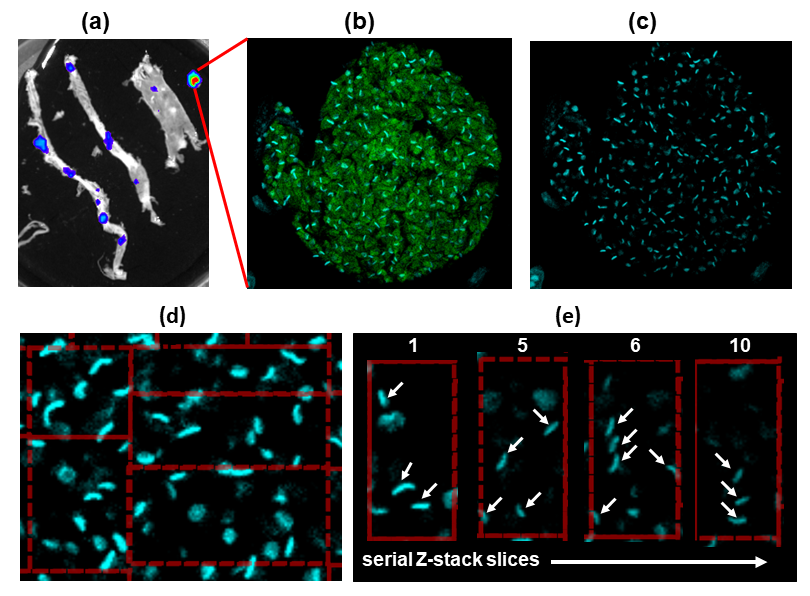

Supplement: FIG S1 [file mBio.01242-20-sf001.tif]

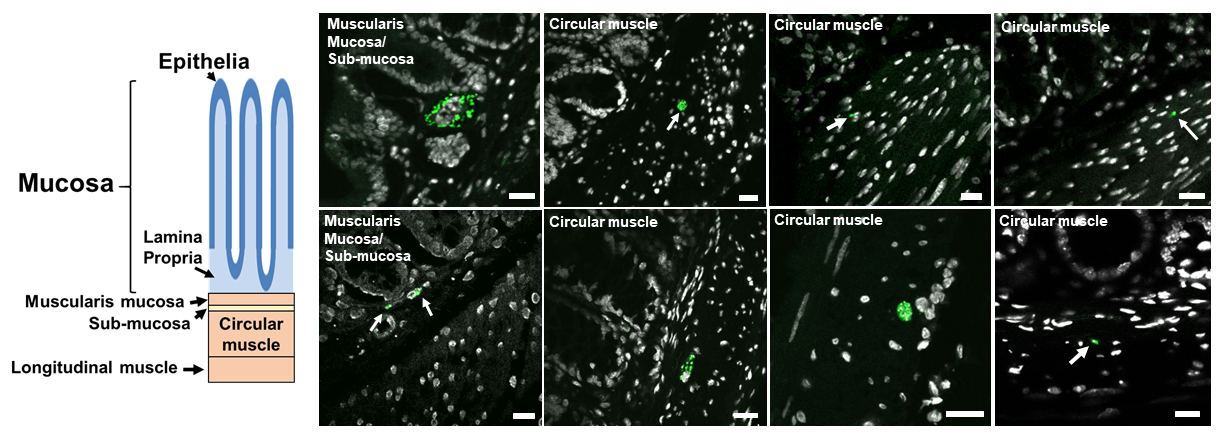

Supplement: FIG S2 [file mBio.01242-20-sf002.tif]
